# Supplementary material for: Diminished circadian and ultradian rhythms of human brain activity in pathological tissue in vivo
Source: Nat Commun. 2024 Oct 2;15:8527. doi: 10.1038/s41467-024-52769-6 (PMC11447262; doi:10.1038/s41467-024-52769-6)
Supplement: Supplementary file 1 — Supplementary Information [file 41467_2024_52769_MOESM1_ESM.pdf]

Supplementary

S1 Imputation of missing data

To facilitate further analysis, we employed imputation techniques to fill any gaps in the relative log band power of each ROI before proceeding with rhythm extraction at various timescales. However, we did not utilise imputed data for the final analysis. We identified missing blocks within each frequency band and imputed them accordingly. If a missing block had a size equal to one, we replaced it with the mean of the value before and after the block. For missing blocks larger than one, we identified the surrounding segments of equal length before and after the block. When the preceding segment was smaller than the missing block or the missing blocks were at the start of the recording, we used only the following segment for imputation. We interpolated the data of missing blocks using the mean of the adjacent segments and added Gaussian noise with a mean of zero and standard deviation of 60% of the standard deviation of the surrounding segments. Any missing data present in the adjacent segments was disregarded. The final values used for analysis were the interpolated ones with added Gaussian noise.

S2 AUC distributions across rhythms and EEG bands

Table S2.1: Almost all AUC distributions are signi cantly greater than 0.5. Shows the T statistic and p value of a one-sided Wilcoxon signed rank test applied to test whether each distribution is greater than 0.5 for each EEG band/biological rhythm pair. No correction for multiple comparisons has been performed.

	Wilcoxon rank sum test [T, p]				
Rhythm	Delta	Theta	Alpha	Beta	Gamma
1h-3h	[631, <0.001]	[538, <0.001]	[681, <0.001]	[662, <0.001]	[590, <0.001]
3h-6h	[632, <0.001]	[486, 0.008]	[648, <0.001]	[649, <0.001]	[551, 0.004]
6h-9h	[630, <0.001]	[568, 0.001]	[590, <0.001]	[630, <0.001]	[581, <0.001]
9h-12h	[628, <0.001]	[493, 0.016]	[510, 0.009]	[550, <0.001]	[551, <0.001]
12h-19h	[576, 0.001]	[460, 0.051]	[490, 0.007]	[561, 0.001]	[511, 0.021]
19h-31h	[498, 0.005]	[479, 0.027]	[492, 0.006]	[594, <0.001]	[596, <0.001]

Table S2.2: Almost all AUC distributions are signi cantly greater than 0.5. Shows the median AUC and the percent of AUCs above 0.5 for each EEG band/biological rhythm pair.

	[median AUC, % $AUC > 0.5$]				
Rhythm	Delta	Theta	Alpha	Beta	Gamma
1h-3h	[0.71, 82%]	[0.64, 74%]	[0.72, 87%]	[0.7, 84%]	[0.71, 74%]
3h-6h	[0.68, 71%]	[0.65, 63%]	[0.68, 82%]	[0.69, 79%]	[0.57, 63%]
6h-9h	[0.69, 76%]	[0.68, 66%]	[0.67, 76%]	[0.75, 71%]	[0.64, 71%]
9h-12h	[0.66, 76%]	[0.64, 63%]	[0.62, 61%]	[0.67, 68%]	[0.62, 74%]
12h-19h	[0.61, 74%]	[0.53, 53%]	[0.61, 55%]	[0.64, 68%]	[0.56, 61%]
19h-31h	[0.6, 71%]	[0.55, 58%]	[0.59, 68%]	[0.67, 71%]	[0.61, 76%]

Table S2.3: Pathology implies diminished rhythms when controlling for brain region. A mixed e ects regression showing the e ect of pathology on rhythm power. Here we show partially standardised coefficient for pathology (SOZ) for each mixed e ects model predicting the cycle power from the brain region of the ROI, and whether the ROI is pathological (in the SOZ). The model is described in section 4.5 of the main text. Each model is built using data from 38 patients.

EEG Band	Rhythm	SOZ Beta	SE	z	pvalue	lower95CI	higher95CI
Delta	1h-3h	-0.301	0.049	-6.127	<0.001	-0.397	-0.205
Delta	3h-6h	-0.257	0.053	-4.864	<0.001	-0.361	-0.154
Delta	6h-9h	-0.17	0.045	-3.821	0.001	-0.258	-0.083
Delta	9h-12h	-0.221	0.051	-4.367	<0.001	-0.32	-0.122
Delta	12h-19h	-0.193	0.053	-3.636	0.001	-0.297	-0.089
Delta	19h-1.3d	-0.185	0.057	-3.269	0.005	-0.296	-0.074
Theta	1h-3h	-0.274	0.063	-4.369	<0.001	-0.396	-0.151
Theta	3h-6h	-0.181	0.048	-3.76	0.001	-0.276	-0.087
Theta	6h-9h	-0.173	0.049	-3.517	0.002	-0.27	-0.077
Theta	9h-12h	-0.171	0.056	-3.037	0.01	-0.281	-0.06
Theta	12h-19h	-0.173	0.052	-3.296	0.005	-0.276	-0.07
Theta	19h-1.3d	-0.129	0.055	-2.329	0.067	-0.238	-0.02
Alpha	1h-3h	-0.262	0.072	-3.662	0.001	-0.402	-0.122
Alpha	3h-6h	-0.257	0.065	-3.959	<0.001	-0.384	-0.13
Alpha	6h-9h	-0.196	0.058	-3.352	0.004	-0.311	-0.081
Alpha	9h-12h	-0.188	0.073	-2.57	0.037	-0.331	-0.045
Alpha	12h-19h	-0.191	0.068	-2.836	0.018	-0.324	-0.059
Alpha	19h-1.3d	-0.102	0.07	-1.462	0.344	-0.238	0.035
Beta	1h-3h	-0.321	0.062	-5.149	<0.001	-0.444	-0.199
Beta	3h-6h	-0.241	0.056	-4.317	<0.001	-0.35	-0.132
Beta	6h-9h	-0.153	0.051	-3.026	0.011	-0.253	-0.054
Beta	9h-12h	-0.223	0.053	-4.185	<0.001	-0.327	-0.118
Beta	12h-19h	-0.194	0.051	-3.838	0.001	-0.293	-0.095
Beta	19h-1.3d	-0.236	0.058	-4.052	<0.001	-0.35	-0.122
Gamma	1h-3h	-0.181	0.037	-4.847	<0.001	-0.254	-0.108
Gamma	3h-6h	-0.156	0.044	-3.582	0.002	-0.241	-0.071
Gamma	6h-9h	-0.186	0.04	-4.599	<0.001	-0.266	-0.107
Gamma	9h-12h	-0.198	0.042	-4.68	<0.001	-0.281	-0.115
Gamma	12h-19h	-0.205	0.046	-4.487	<0.0010.0	-0.294	-0.115
Gamma	19h-1.3d	-0.216	0.051	-4.201	<0.001	-0.317	-0.115

S3 Diminished rhythms and seizure load

In the main paper we show than the diminished rhythms is consistent in time, without dependence on seizure timing. To provide further evidence that seizure load is not driving the result, we here show (figure S3.1 the correlation between seizure load and AUC. We found either no correlation or weak correlation between seizure load and AUC.

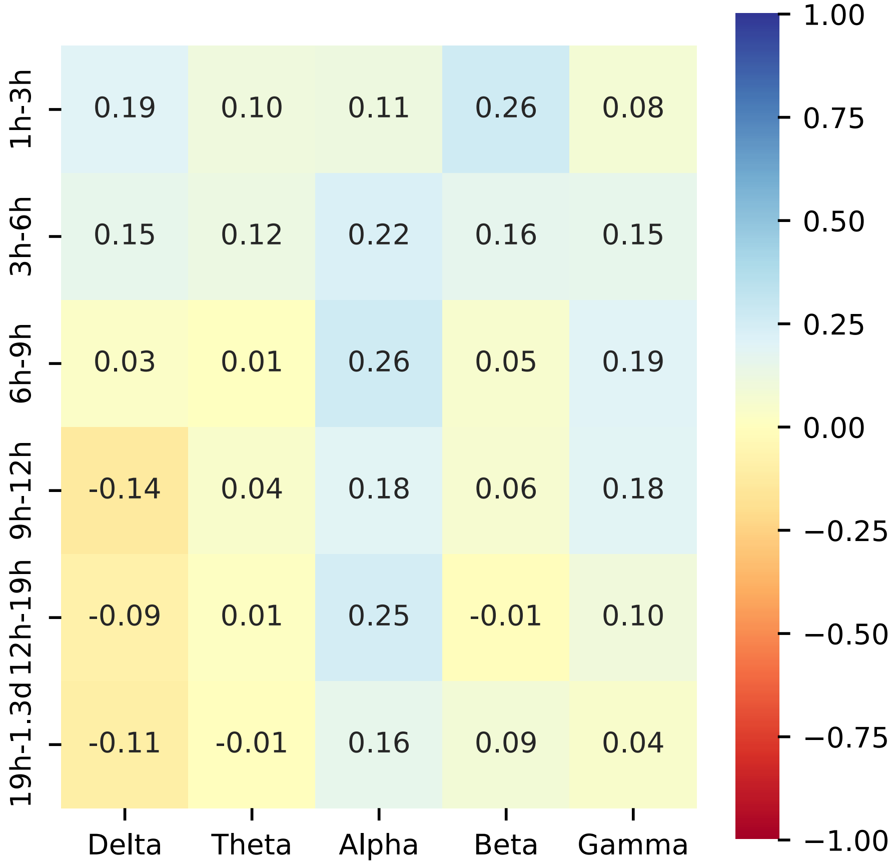

Figure S3.1: There are no strong correlations between seizure load and diminished chronobiological rhythms. The seizure load was calculated as the number of seizures per hour. A Pearson correlation was calculated between this and the AUC for each ultradian and circadian rhythm and EEG band. All coefficients were below 0.3 (with most below 0.1), indicating that in most cases there was no correlation and at most a weak correlation. Above figure shows the coefficients (r) for each combination. For each correlation n=38.

S4 Signal magnitude in the SOZ

We wished to rule out the possibility that di erences in the power of the signal between the SOZ and the rest of the tissue may be driving the diminished rhythms we see. To investigate this we have summed the raw band power values in all frequency bands and across time to obtain the overall signal power in each ROI (equation 2). Where T is the number of samples, δBP is a (T x nROI) matrix containing the power of the signal in the delta band, and TotalPowerROI is a vector containing the total power of the signal in each ROI. We then calculated an AUC with the $TotalPowerROI$ in ROIs within the SOZ against the $TotalPowerROI$ in ROIs not in the SOZ to get an AUC_{TP}.

$$TotalPowerROI = log \quad \sum_{t=}^{t=T} \delta BP_t + \theta BP_t + \quad BP_t + \beta BP_t + \gamma BP_t \Bigg) \tag{2}$$

$$AUC_{TP} = AUC\left(TotalPowerROI[SOZ], TotalPowerROI[notSOZ]\right) \tag{3}$$

We first tested whether the log of the overall signal power di ered in pathological tissue (SOZ) compared to other tissue (Figure S4.1 a). In terms of percentage di erence between pathological and other tissue, there was a wide spread across the cohort from -20% to +20%, and the median as approx +3% indicating little to no di erence in overall signal power between tissue types on average. If at all, the pathological tissue may show elevated signal power across the cohort, possibly due to interictal spike or similar. We also tested if overall signal power distinguishes pathological tissue (SOZ) from other tissue using the AUC_{TP} (Figure S4.1 b). Over the cohort, again, there was a slight shift towards pathological tissue displaying higher overall signal power than other tissue, again, possibly due to interictal phenomena.

We then directly tested if AUC_{TP} correlated with the AUC values we obtained in the main results (using the relative band powers in each band). Our results show that this is not the case, most correlations are very weak (< 0.2), and all correlations are < 0.4 (Figure S4.2). We conclude that the chronobiological rhythms seen in relative band power are not directly determined by overall signal magnitude, i.e. the reported e ects are not simply due to a weaker EEG signal in pathological tissue.

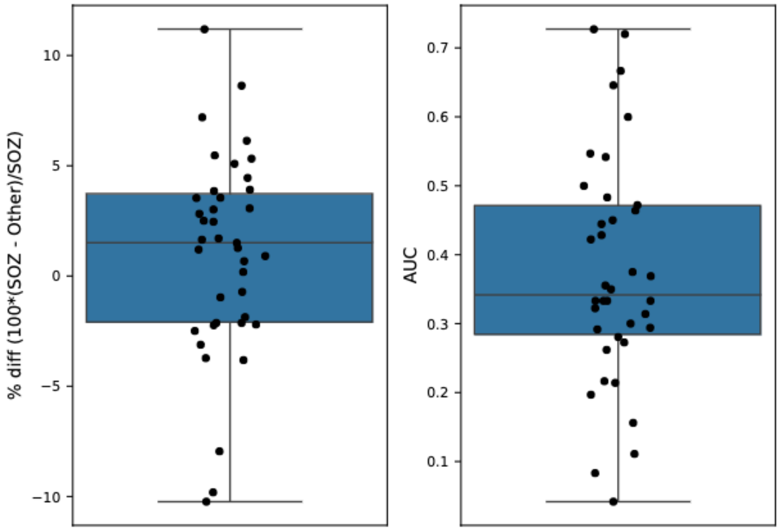

Figure S4.1: (A) The di erence between the raw log power in the SOZ and non-SOZ. (B) Using the raw log power to calculate an AUC in the same manner as we calculated AUCs for the power of the chronobiological rhythms.

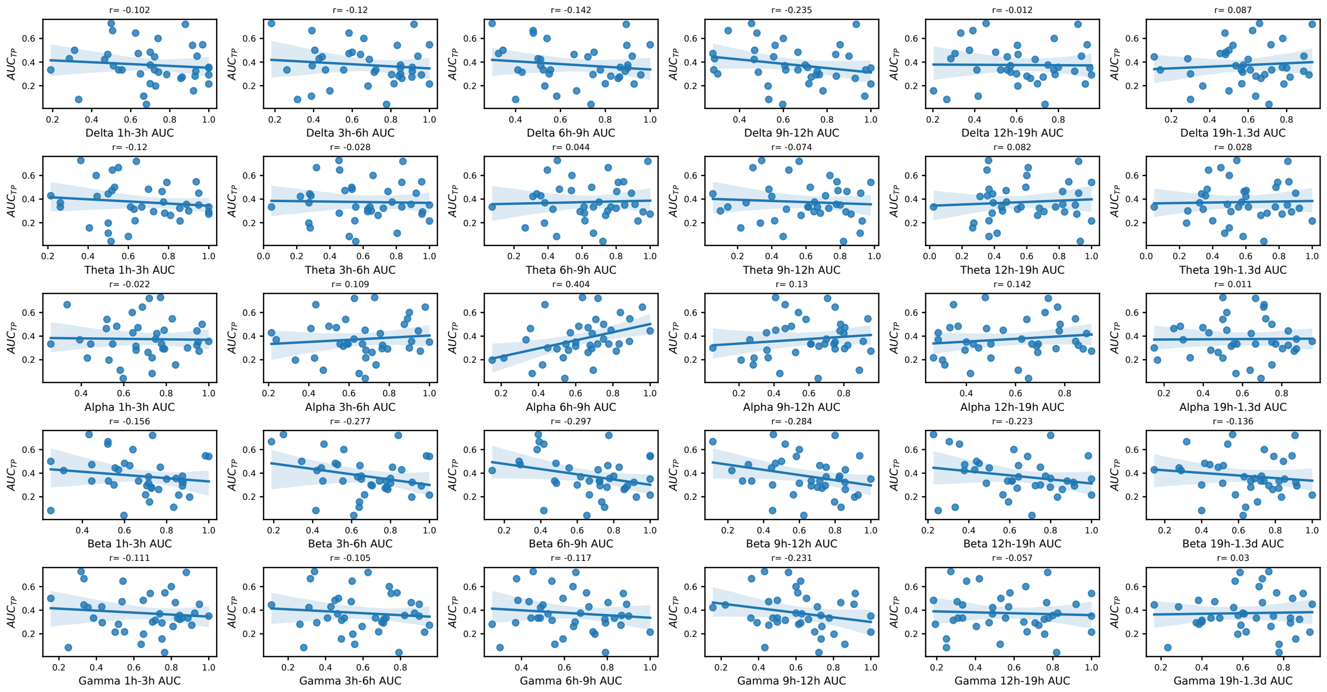

Figure S4.2: The correlation between the raw power and the AUC calculated on the chronobiological rhythm for each EEG band. For delta, theta, and alpha, we see no correlation or minimal correlation (r<0.3). For beta and gamma moderate negative correlations are present.

S5 Cycles are also diminished in resected *vs* spared tissue

In the main analyses of this paper we have used the seizure onset zone (SOZ) to indicate whether pathology is present in a region. An alternative marker is whether the tissue was resected during surgery. To assess whether chronobiological rhythms remained diminished when using a resection as a marker for pathology, we re-calculated AUC values for each rhythm and EEG band using this instead of SOZ, and present them above. For delta, theta, alpha, and beta, we find median AUC values above 0.5 for most rhythms - similar to the results using SOZ and indicating diminished rhythms in resected tissue. For gamma, we median AUC values around 0.5 - indicating no di erence in the strength of the chronobiological rhythms between resected and spared tissue.

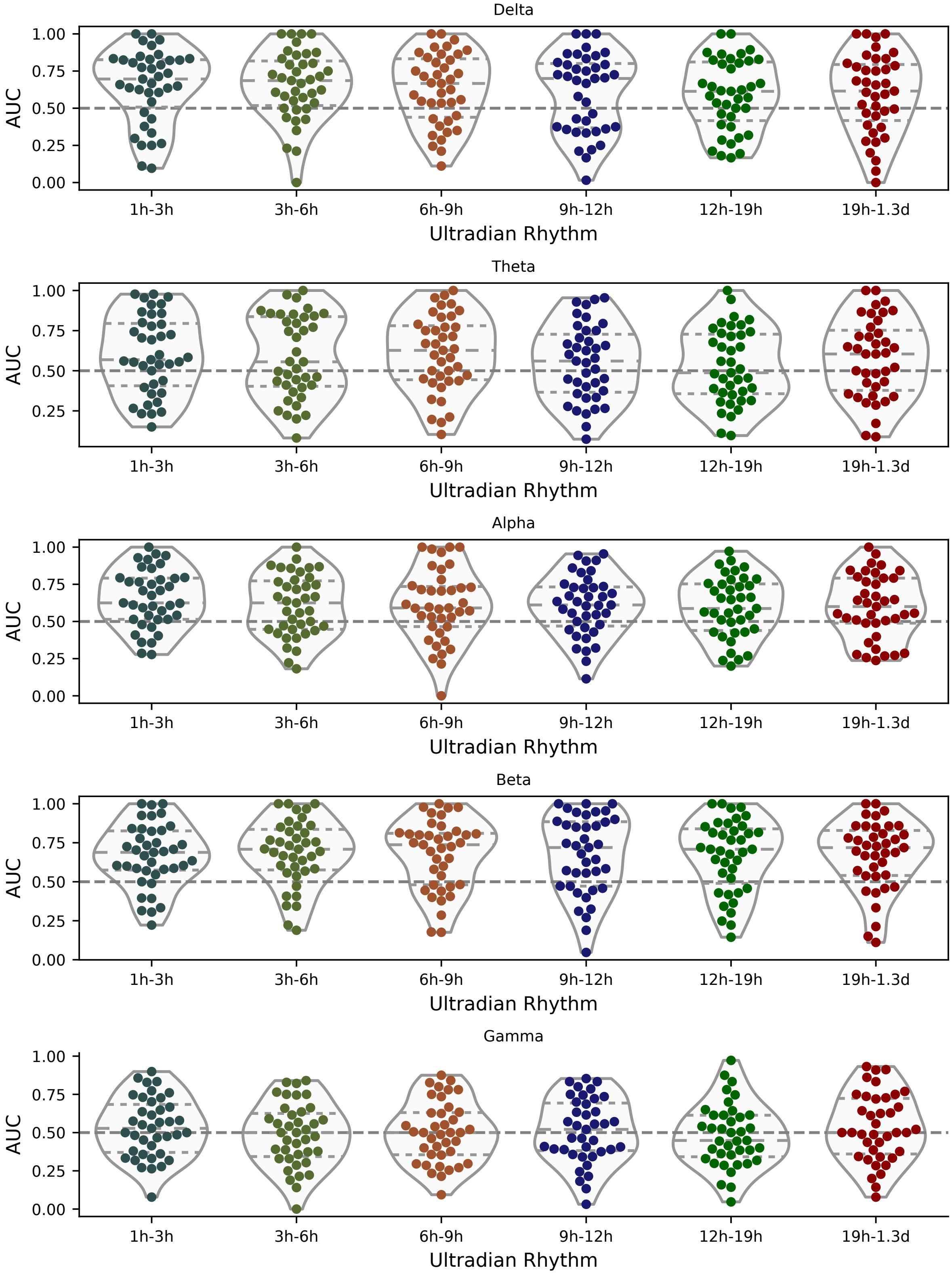

Figure S5.1: The AUC values calculated on resected regions against spared regions for each patient in each chronobiological rhythm and each EEG band.

S6 Cycles are diminished in multiple types of pathology

Figure S6.1 shows the AUC of distinguishing pathological tissue from other tissue in terms of magnitude in the circadian and one example ultradian rhythm in relative delta band power. Our sample was too small to draw definitive conclusion on any individual pathology, but the most common pathologies of hippocampal sclerosis (HS) and focal cortical dysplasia (FCD) both demonstrate the diminished rhythmicity. Also most subjects show this e ect, without sufficient evidence for an influence of pathology type.

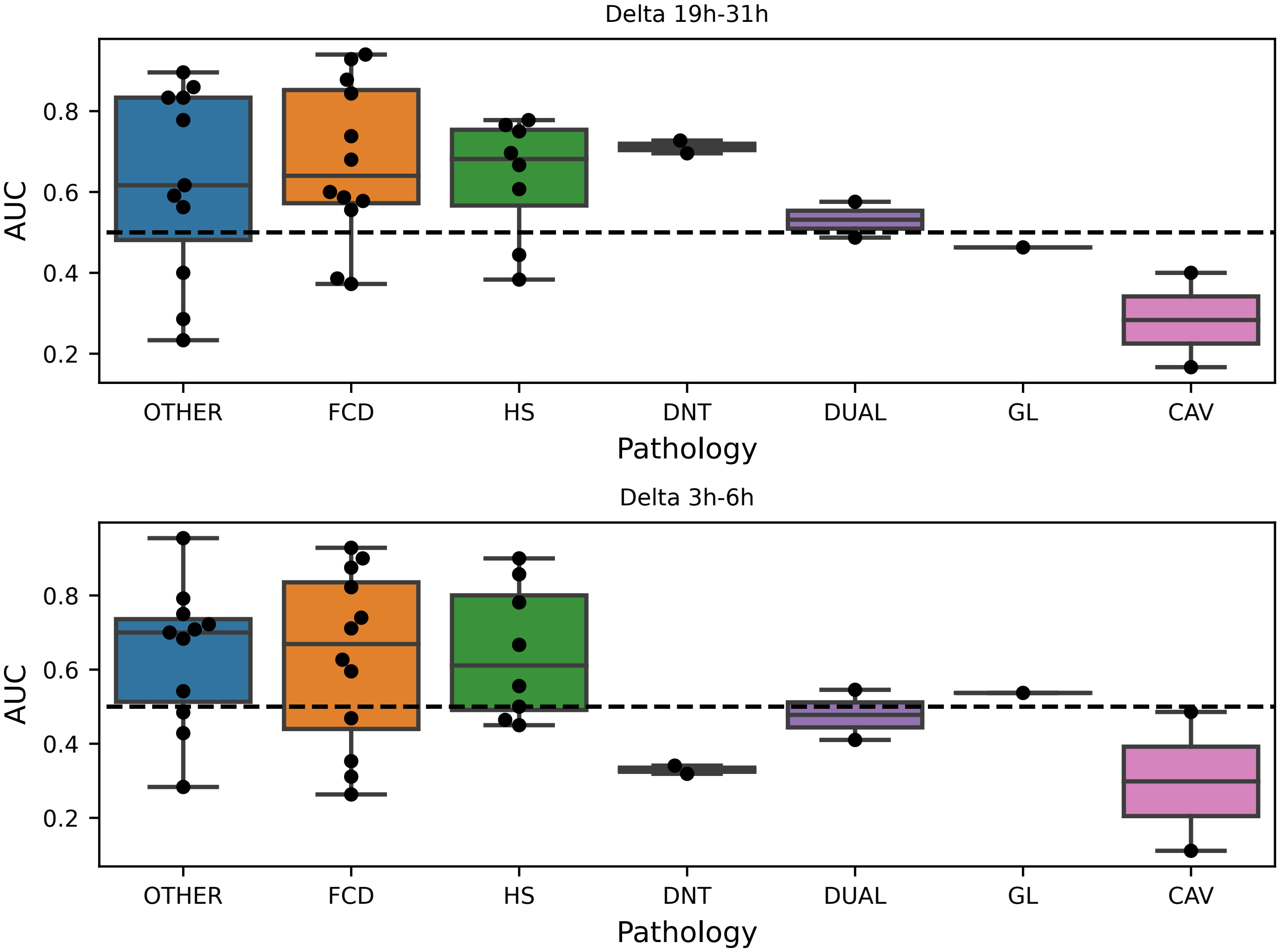

Figure S6.1: Top: AUC values of distinguishing pathological tissue *vs* other tissue in terms of the circadian rhythm magnitude in relative delta band power plotted by type of pathology. Bottom: Same as top, but for the 3-6h ultradian rhythm. Pathology abbreviations: hippocampal sclerosis (HS); focal cortical dysplasia (FCD); dysembryoplastic neuroepithelial tumor (DNT); gliosis (GL); cavernoma (CAV); multiple pathologies (DUAL), other pathologies (OTHER)

S7 Diminished rhythms across age, sex, epilepsy type, and anti-seizure medication

Overall, we did not observe noteworthy modulatory/differential effects of age, sex, epilepsy type, or anti-seizure medication on the diminished power of the circadian rhythm in pathological tissue (Fig. S7.1 and Fig. S7.2). We did observe a moderate difference (with $AUC = 0.3$ and $p = 0.04$) between TLE and eTLE in terms of their AUC distinguishing signal power between pathological and other regions. However, upon further investigation by including a categorical variable into out mixed effect model, we did not see any substantial or significant effects on TLE vs eTLE. Therefore, the moderate effect seen here is most likely driven by the different regions implanted in TLE vs eTLE.

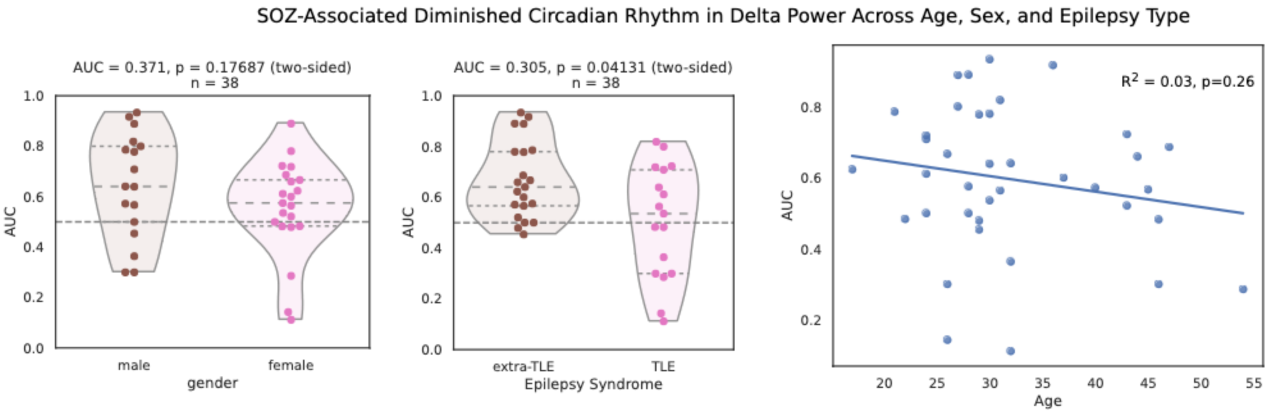

Figure S7.1: AUCs indicating the diminished power of the circadian rhythm in delta power in the SOZ, comparing patients split by Sex, Epilepsy Type (TLE or eTLE), and age. We saw no significant di erence with regards to sex, but the rhythm power was more diminished in eTLE compared to TLE (p=0.04). A negative slope with found in the regression with age, however this was weak ($R^2 = 0\ 03$).

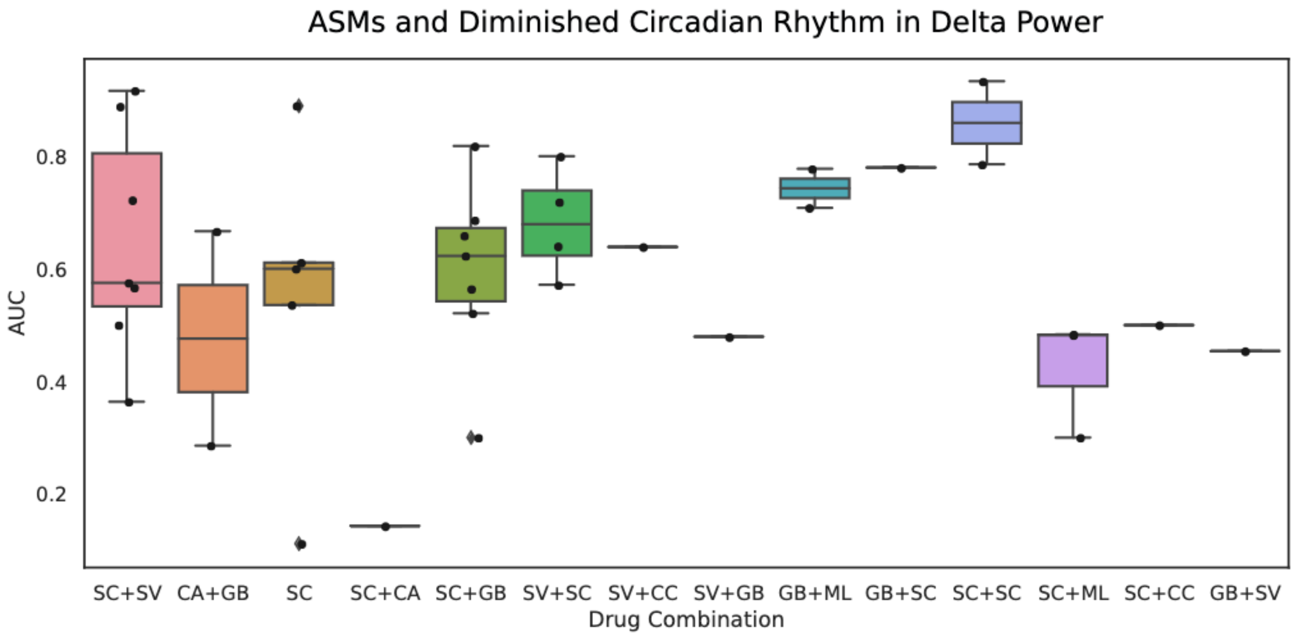

Figure S7.2: AUC indicating the diminished power of the circadian rhythm in delta power in the SOZ, comparing the anti-seizure medication (ASM) combinations the patients were using at the time. Due to the large variation in drugs used, and the multiple combinations of drugs, we could not draw any conclusions from this data. Drug type is indicated by its mechanism of action, with SC: sodium channel blocker (e.g. Carbamazapine, Lamotrigine), GB: GABA enhancer (e.g. Clonazepam, Diazepam), CC: calcium channels (e.g. Gabapeptin), GT: glutamate receptors (e.g. Perampanel), SV: SV2a receptor (e.g. Levitiracitam), CA: inhibition of carbonic anhydrase (e.g. Acetazolamide), ML: multiple targets (e.g. Sodium Valproate).

S8 Using a tighter definition of circadian rhythms

In the main text we use a broad definition of circadian rhythms as fluctuations with a period of between 19 and 31 hours, and parameterise our band pass filter accordingly. To investigate whether a narrower definition of the circadian fluctuations we recalculate them using a definition of 20-26 hours. Figure S8.1 replicates figure 1 (a and d) from the main text, using the narrower definition. We find that for our example subject we get a similar pattern of signal power across regions, and we get a similar overall AUC of 0.917. Figure S8.1 (b) shows that across the population we get a very similar distribution of AUCs, with 0.6 as the median.

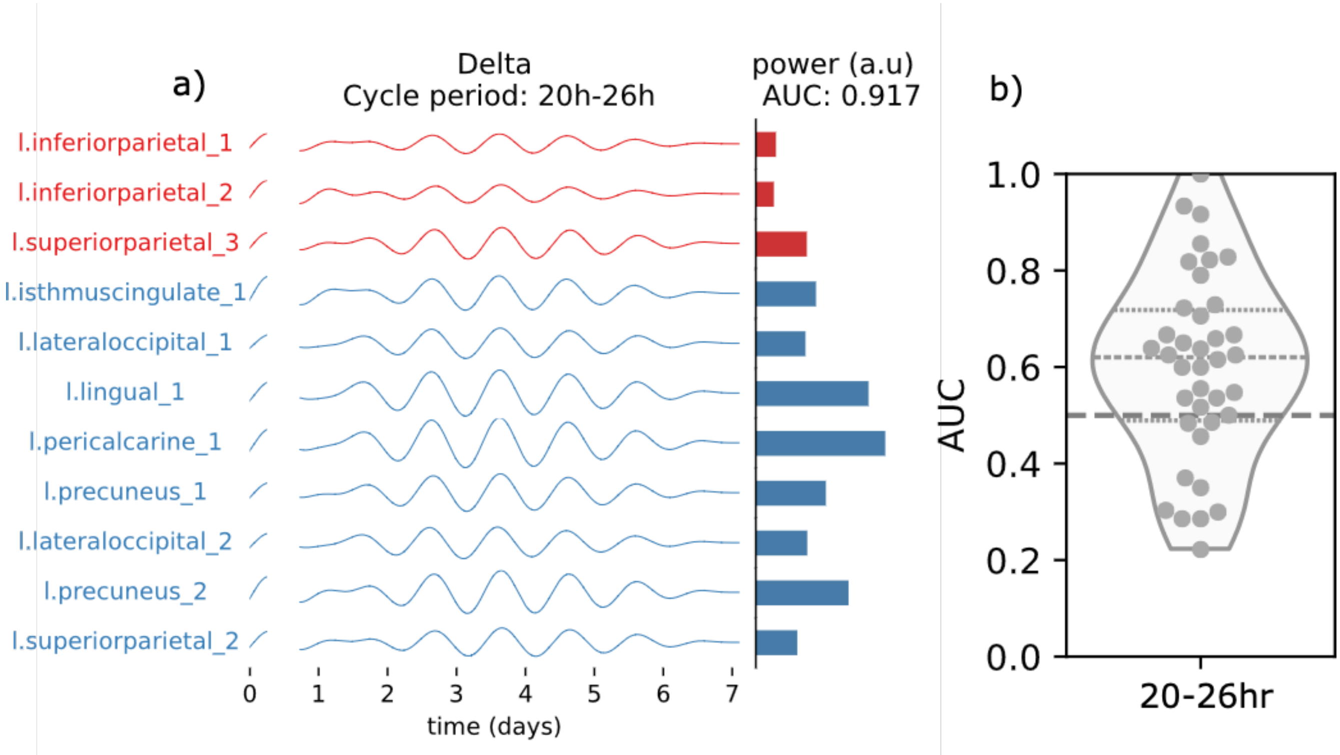

Figure S8.1: Circadian rhythms are diminished in the SOZ to a similar extent when using a narrower definition. a) Shows the power of delta oscillations in each region, filtered to isolate the circadian component (with a period of between 20 and 26 hours) for the example subject used in figure 1 in the main text. Red lines indicate regions that are pathological (within the SOZ). This subject has an AUC of 0.917 indicating that the magnitude of the rhythm is diminished in the pathological areas. b) shows the value of AUCs for all patients, most are above 0.5 indicating that the rhythm is diminished in the SOZ for most of the cohort.

S9 Circadian patterns of seizure occurrence

While this study focussed on the circadian and ultradian rhythmicity in the power of the canonical frequency bands in the iEEG, it is well known that seizures themselves can occur at particular phases in these rhythms for some patients. To investigate further whether this played any role in the diminished rhythms we have found in the SOZ, we show here the phase locking value of seizures to the 24 hour daily cycle, the filtered circadian cycle in delta power in the pathological regions, and in the other regions. Phase locking value (PLV) here is calculated following previously outlined methods (44), where a PLV of 1 indicates all seizure occur at the same phase, while 0 indicates a uniform distribution of phases. We found that for the majority of patients the phase locking values did not imply strong phase preference for any of the three cycles. In a minority of patients phase preference did exist, for the 6 patients with the strongest preference in figure S9.1 (b) we show the mean rAUC across the 24 hour cycle and the phase of each seizure. In none of these cases do we see changes in the rAUC at the phase preference of the seizures, emphasising that rhythms are diminished independently of seizure occurrence.

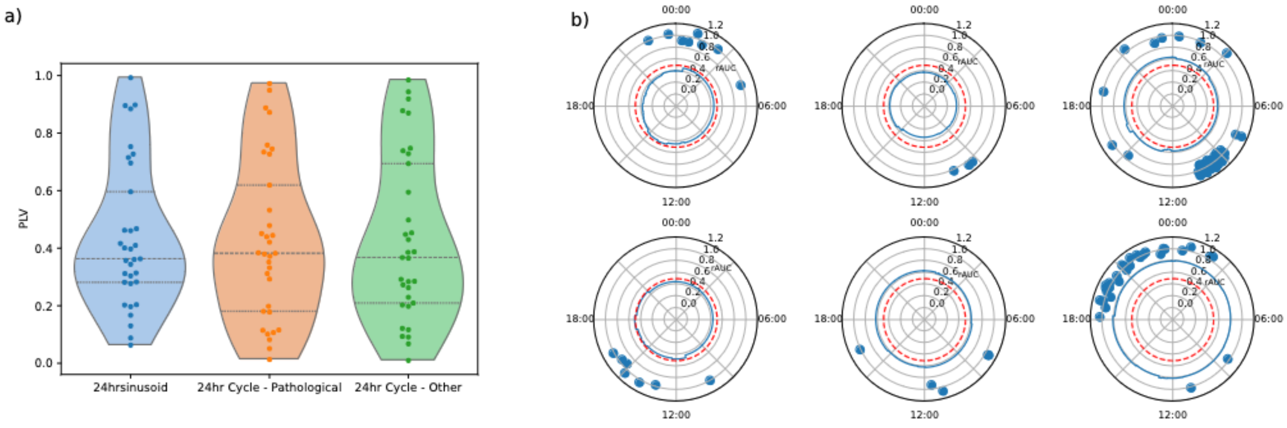

Figure S9.1: a) Shows the distribution of phase locking values across the cohort quantifying the phase locking of their seizures to the 24 daily clock cycle, the circadian cycle of their delta power in pathological brain regions and their circadian cycle in delta power in their other brain regions. b) Shows the timings of seizures in the 24 hour cycle for the 6 patients with the highest phase-locking value - blue circles. The rolling AUC (rAUC) is plotted in blue with the dashed red line indicating an AUC of 0.5.

S10 AUCs in the post-ictal period

While we show the AUC values in the peri-ictal period in figure 4, the post-ictal period - 30 minutes post seizure - is also known to show aberations in the EEG. Here we calculated an rAUC value solely based on the power of the circadian rhythm during this 30 minute period, shown in figure S10.1. We find that the AUC values here are similar to those presented in figure 4.

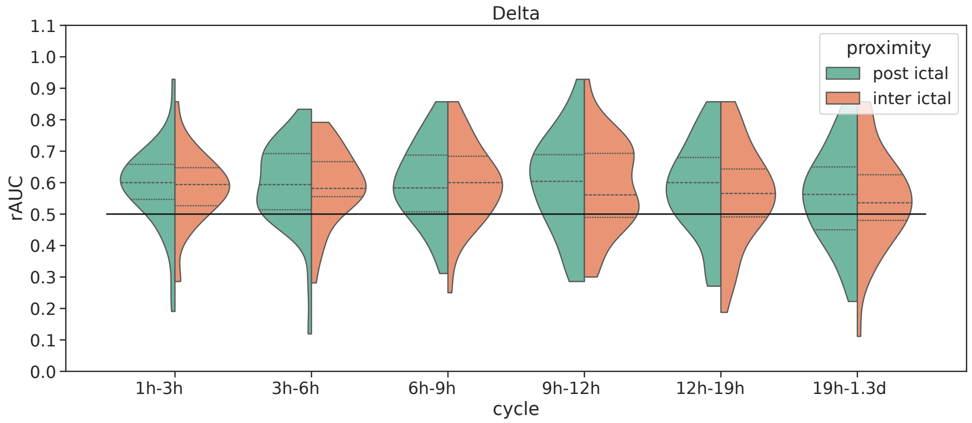

Figure S10.1: Shows the rAUCs calculated using only the power of the daily rhythm in delta power in the 30 minutes post-seizure.

S11 Changes in daily rhythm power over time

The amplitude of the biological rhythms does not remain constant across the recording period for most patients. To investigate this we captured the amplitude of the daily rhythm in delta power over time for each patient by finding the peaks of the absolute value of the band pass filtered signal. We did this for signals within the SOZ and for those in other brain regions. This gave us the lines shown in figure S11.1. We then assigned these to three categories - those that show a clear amplitude loss over time (SOZ n=12, other n=10); those that fit well to a Gaussian curve, with a peak in the middle of the recording and lower amplitudes at the edges (SOZ n=13, other n=15); and those that do not meet either of these criteria (SOZ n=14, other n=14).

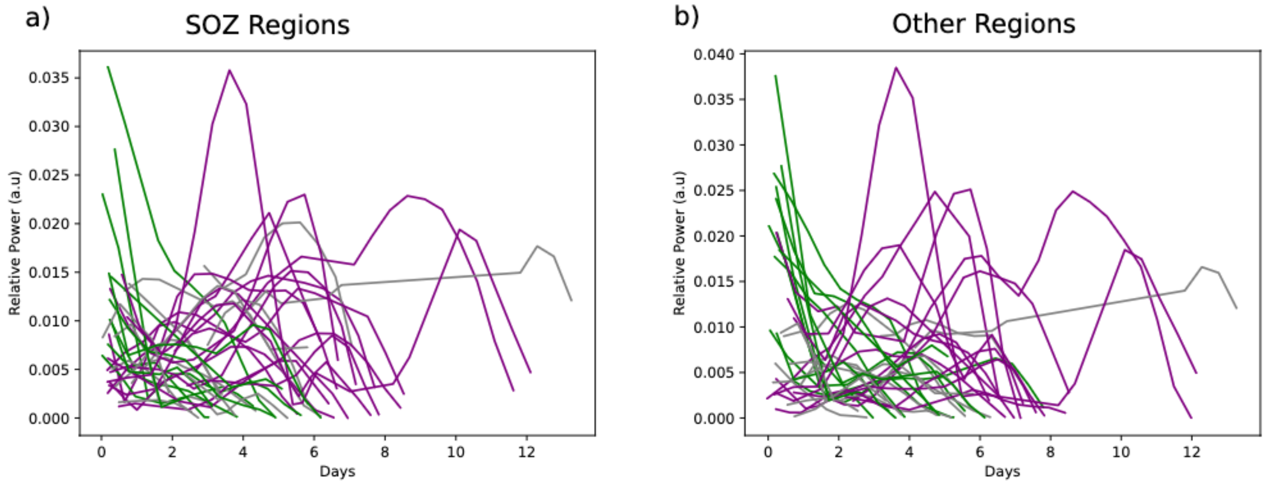

Figure S11.1: Shows the amplitude of the daily (circadian) rhythm in delta power over time for each of the patients, in the SOZ (a) and in other regions (b). Colors correspond to three categories. Green - clear decrease in amplitude (the maximum peak occurs in the first three peaks), purple - fits well to a Gaussian curve ($p < 0\ 01$ in a one-sided Kolmogorov-Smirnov test), and the remainder of patients.
